# Tethered Balloon-Borne Turbulence Measurements in Winter and Spring during the MOSAiC Expedition

**DOI:** 10.1038/s41597-023-02582-5

**Published:** 2023-10-19

**Authors:** Elisa F. Akansu, Holger Siebert, Sandro Dahlke, Jürgen Graeser, Ralf Jaiser, Anja Sommerfeld

**Affiliations:** 1 Leibniz Institute for Tropospheric Research, Leipzig, Germany; 2 Alfred Wegener Institute Helmholtz Centre for Polar and Marine Research, Potsdam, Germany; 3 Present Address: Forschungsverbund Berlin e.V., Berlin, Germany

**Keywords:** Atmospheric science, Atmospheric dynamics

## Abstract

During the Multidisciplinary Drifting Observatory for the Study of Arctic Climate expedition, a tethered balloon system was operated with a turbulence probe attached to study the lower troposphere in the high Arctic. Overall, measurements were conducted on 34 days between December 2019 and May 2020, resulting in 47 quality-assured sampling records consisting of vertical profiles and constant-altitude measurements. The continuous profiles extend from the surface, i.e., the sea ice floe, to a height of several hundred meters typically. The high-resolution wind velocity measurements using a hot-wire anemometer and temperature measurements using a thermocouple provide a comprehensive basis for examining the dynamical processes and thermodynamic stratification in the Arctic atmospheric boundary layer under cloudless and cloudy conditions. This paper provides a detailed technical description of the turbulence payload, including calibration and quality assurance, and a general overview of the data. A particular focus of this work is the estimation of local energy dissipation rates. The data are freely available from the World Data Center PANGAEA.

## Background & Summary

Global warming is steadily progressing, and its effects are clearly visible, especially in the Arctic, with a reduction in sea ice. Near-surface temperatures in the Arctic have significantly increased in recent decades, with warming rates more than twice the global warming value^1^. This phenomenon is widely known as Arctic amplification and is the most pronounced in winter^1–3^. Several feedback mechanisms contribute to Arctic amplification, with surface albedo feedback and lapse rate feedback considered the main drivers^4^. Furthermore, the often persistent and stratiform low-level clouds play an essential role due to their notable influence on the surface energy budget^5^. Particularly in winter, the Arctic atmospheric boundary layer (ABL) is often very shallow and characterized by very stable conditions, especially under cloudless conditions. The sea ice surface and radiative cooling favor the evolution of surface-based temperature inversions. Near-surface inversions also fulfil a significant role in the Arctic climate system, as they are key drivers of the aforementioned lapse rate effect^6^.

Turbulence plays an important role in vertical exchange between different layers, although it is typically quite low under these stable conditions. One important source of turbulence occurs within the context of clouds and results from radiation-induced cooling at the cloud top with subsequent mixing^7^. Clouds alter the radiative and turbulence processes in the ABL, often leading to the development of a relatively well-mixed ABL not only in summer but also in winter. This mixing, which originates from the cloud layer, however, does not always extend to the surface, leading to decoupling of the subcloud layer from the surface. The ABL usually alternates between two typical states: near-surface temperature inversions under cloudless conditions with wind shear-induced near-surface turbulence and a more mixed neutral ABL under cloudy conditions capped by an elevated inversion above the cloud top^8^.

The central Arctic is a harsh environment with limited access, especially in winter and spring. Although observations in these remote areas are generally difficult, ship expeditions at high latitudes are regularly conducted, but mainly in summer and limited to shorter passages, and only very few observations in winter are available^3,9^. However, to improve the understanding of the Arctic climate system and its turbulent atmospheric processes, *in situ* observations in the central Arctic are needed throughout all seasons. The Multidisciplinary Drifting Observatory for the Study of Arctic Climate (MOSAiC) expedition, drifting with an ice floe, achieved this goal from October 2019 to September 2020. The MOSAiC expedition provided a unique opportunity to measure many parameters of the coupled Arctic climate system, including the ocean, sea ice, and atmosphere over an entire annual cycle^10–12^.

From winter 2019 to spring 2020, the helium-filled tethered balloon *Miss Piggy* of the Alfred Wegener Institute was operated on sea ice with various payloads^12^. The balloon setup is modular and allows the study of different scientific questions^13^. However, the focus of this paper is data obtained with a turbulence probe to measure small-scale turbulence in the ABL. Other systems operated on the balloon, such as an aerosol filter sampler analyzing ice-nucleating particles, an optical particle counter measuring the aerosol particle size distribution, and an ozone measurement device targeting the regions in and above the ABL, are not considered in this paper, and the reader is referred to the overview paper^12^.

**Fig. 1:**
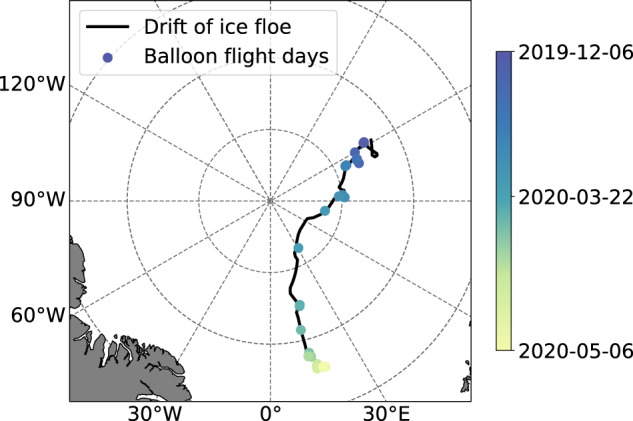
Location of RV *Polarstern* and the ice floe during the measurement period of the tethered balloon *Miss Piggy* and the turbulence probe. Days of flight operation are indicated by dots.

**Fig. 2:**
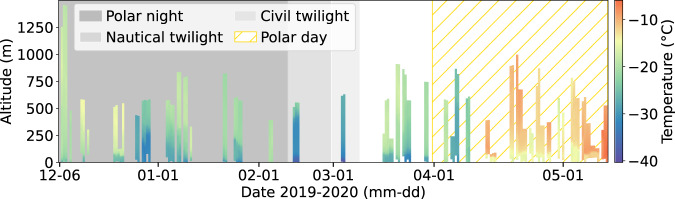
Overview of all profiles, their maximum altitude reached, and the temperature measured using the turbulence probe during the MOSAiC expedition (legs 1, 2, and 3). The respective daylight condition during the operation period is indicated by shading.

The turbulence probe consists of a fast-responding hot-wire anemometer, thermocouple, and standard meteorological instruments for thermodynamic and dynamic properties^14^. This setup allows measurements of the horizontal wind velocity with a high temporal resolution of 125 Hz and temperature with a reduced sampling frequency of 10 Hz. The measurements of the turbulence probe cover the period from 6 December 2019 to 6 May 2020 between 86.14° N 122.21° E and 83.92° N 17.69° E (Fig. 1). A total of 47 sampling measurements (about 67 flight hours) were performed on 34 days. Overall, the operations during the MOSAiC expedition provided large numbers of vertical profiles of the lower atmosphere plus measurements at a certain height during the polar night and the transition to the polar day. Figure 2 shows an overview of the balloon deployments, temperature, and respective daylight conditions. This dataset provides detailed insight into the thermodynamic stratification and turbulent properties of the Arctic ABL under cloudless and cloudy conditions. Our tethered balloon turbulence observations of the ABL are limited to legs 1 to 3 for technical reasons; however, especially during the following leg 4, intensive turbulence measurements in the ABL were also conducted with UAVs and tethered balloons, which we refer to here^12,15^.

In the following *Methods* section, the calibration of the high-resolution turbulence sensors using a reference probe on the balloon and the determination of the local energy dissipation rate *ε* as a local turbulence parameter are described. A general discussion of the data quality and possible problems in data interpretation is given in the *Technical Validation* section, together with measurement examples to ensure widespread use of the presented data.

## Methods

### Tethered balloon-borne measurements and operations

During the MOSAiC expedition, the 9 m^3^ helium-filled tethered balloon *Miss Piggy* was launched from Balloon Town on the ice floe near the research vessel *Polarstern*^16^, which drifted with the ice floe^12^. During the deployment of the turbulence probe (legs 1 through 3), Balloon Town was located at a safe distance of approximately 250 to 380 m from RV *Polarstern* and was equipped with a tent to prepare the instruments before launch. Furthermore, a meteorological tower, hereafter referred to as the Met Tower, was operated in Met City, used to conduct continuous meteorological measurements at heights of 2 m and 10 m^17^. Details on the floe layout and the continuous and periodical measurements throughout the MOSAiC expedition can be found in Shupe *et al*.^12^.

The tethered balloon allows *in situ* observations between the surface and 1500 m, even in clouds and under light icing conditions^13,14^. The balloon enables only lightweight measuring platforms to be lifted since its lifting capacity is limited to a few kilograms depending on wind conditions. Weather conditions restrict balloon operation; here, heavy precipitation with possible icing but mainly wind conditions are decisive. The wind velocity at the surface should not be much higher than 5 m s^−1^ and should not exceed 15 m s^−1^ at altitude. These limitations, together with general restrictions due to other measurement interests of the expedition, automatically lead to biased sampling, which must be considered in data interpretation.

During the deployment period, a Vaisala tethersonde TTS111^13^, hereafter referred to as the tethersonde, and the above turbulence probe were mounted along the tether of *Miss Piggy*. The instruments were attached to the tether below the balloon, with the tethersonde always closest to it at a distance of approximately 10 m. The turbulence probe can be fastened at different points on the tether. Depending on the sampling setup, the distance between the tethersonde and the turbulence probe can vary between approximately 10 and 20 m.

An electric winch allows continuous ascent or descent with a constant climb speed of approximately 1 m s^−1^. The duration of a given profile mainly depends on the maximum altitude reached. For example, the typical sampling time of a profile up to a height of approximately 600 m is approximately 10 minutes. In addition, the balloon can be held at a constant altitude for sampling over a certain period of time, which was done occasionally. However, the constant-altitude sampling legs were almost always above the mixing layer height because other scientific questions were pursued there.

### Hot-wire anemometer package

The hot-wire anemometer package, referred to as the turbulence probe, is designed to measure turbulence using a constant-temperature hot-wire anemometer with a sampling frequency of *f*_*s*_ = 125 Hz and was further developed relative to Egerer *et al*.^14^. A fast-responding thermocouple was used to measure the temperature (*f*_*s*_ = 10 Hz). In addition, a Prandtl tube is installed as a reference for wind velocity measurements, and a PT100 as a temperature reference. The MSR-160 data logger (Modular Signal Recorder, MSR Electronics GmbH) used includes fast analog inputs for the hot-wire anemometer and thermocouple and slow sensors with a resolution of one second for determining static pressure, temperature, and relative humidity.

**Fig. 3:**
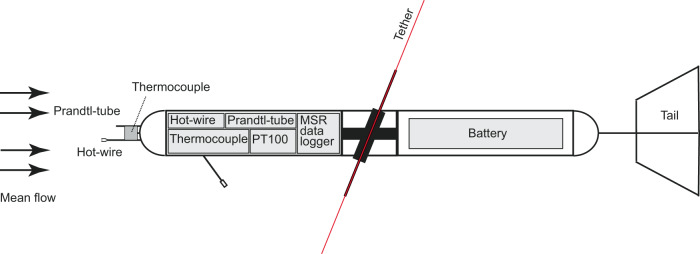
Schematic representation of the hot-wire anemometer package used on the tethered balloon during legs 1, 2, and 3 of the MOSAiC expedition. The red line visualizes the tether of the balloon.

The sensors were installed in a polystyrene body tied to the tether. Figure 3 shows a schematic of the module. All sensors and the data logger occur in the front part of the probe. The probe is fastened centrally, balanced by the weights of the battery pack and the tail unit in the rear. The attachment mechanism of the instrument to the tether allows the turbulence probe to align itself along the mean flow direction due to the torsion of the tether. Furthermore, the probe can perform free pitching movements and is thus independent of the inclination of the tether. A wind vane (tail) ensures alignment of the probe with the mean flow. The orientation to the flow is important to gain reliable wind measurements.

Pressure records are used to calculate the barometric height *z*_*b*_ above the surface (Index “0”) as follows:

1 $${z}_{b}=\frac{{T}_{0}}{\Gamma }\left[1-{\frac{p}{{p}_{0}}}^{\left(\frac{{R}_{d}\Gamma }{g}\right)}\right],$$

with the gas constant *R*_*d*_ = 287.05 J kg^−1^ K^−1^ for dry air, the acceleration due to gravity *g* = 9.81 m s^−1^, and the temperature lapse rate of the standard atmosphere Γ = 0.0065 K m^−1^. The temperature measured by the Met Tower at a height of 2 m is used as the surface temperature. The surface pressure is obtained from the turbulence probe before launching and after landing and linearly interpolated to account for surface pressure changes during the flight. In the case of error-prone data, the tendency of the surface pressure during the flight is derived from the Met Tower. The fine-wire sensors must be calibrated to obtain wind velocity and temperature values. However, the ambient temperature conditions influence the calibration properties, which needs to be considered. Therefore, the data processing and calibration are conducted offline after each flight with simultaneous wind and temperature reference measurements that are recorded with lower frequency^18^.

### Vaisala tethersonde TTS111

A tethersonde (Vaisala TTS111^19^) was attached to the tether during all flights with a radio link to a ground station for online monitoring of atmospheric conditions and flight safety. This type of tethersonde has demonstrated its capabilities under polar conditions during many expeditions^20^. Thermodynamic parameters (static pressure, temperature, and relative humidity) were observed with standard devices of Vaisala radiosondes, with the wind velocity measured using a 3-cup anemometer with a light chopper tachometer. The wind direction can, in principle, be observed with a magnetic compass but not in this study because of the proximity of the magnetic north pole and the associated high uncertainty. The absolute accuracy in sounding is given in the manual^21^ as 0.5 K for temperature measurements, 5% for relative humidity measurements, and 1.5 hPa for pressure measurements corresponding to about 12 m for the barometric altitude. However, the reproducibility during probing is given as 0.2 K, 3%, and 0.5 hPa (4 m), respectively. Heating phenomena influencing the temperature measurements can be neglected during polar night. The manufacturer specifies a resolution of 0.1 ms^−1^ for wind velocity measurements, but no absolute accuracy is given in the manual. However, the wind tunnel tests reported in^19^ indicated a worst-case deviation from a reference wind sensor of 0.3 ms^−1^, which is chosen as the absolute accuracy in this study.

### Calibration of the hot-wire anemometer and thermocouple

A constant-temperature anemometer is based on flow-dependent energy transfer of a hot wire held at a constant temperature *T*_*w*_ to its environment at temperature *T*_*a*_ with *T*_*w*_»*T*_α_. This energy transfer can be formally expressed by the King’s law, which provides the following expression for the fluid velocity *U*:

2 $$U=\frac{1}{{D}^{\frac{1}{n}}\rho }{\left[\frac{{E}_{w}^{2}}{\left({T}_{w}-{T}_{a}\right)}-c\right]}^{\frac{1}{n}}$$

with the output voltage of the hot-wire circuit *E*_*w*_, the air density *ρ*, and empiric parameters *c*, *D*, and *n*^18,22,23^.

As noted by Frehlich *et al*.^18^, the constants in King’s law are not general but may vary with ambient conditions, composition, and age of the wire. Therefore, calibration was conducted individually for each vertical profile and each constant-altitude sampling leg. The parameters *c*, *D*, and *n* were estimated using least-square fitting where *U* and *T*_*a*_ were measured by the tethersonde. As the Prandtl tube was frequently affected by icing, the tethersonde served as a reference. Icing did not affect the cup anemometer measurements of the tethersonde or the hot-wire anemometer. With this method, however, one must consider that both the hot-wire and tethersonde measurements must be performed at the same place and time, which is not possible for technical reasons. Instead, the two instruments were attached to the line with a vertical separation of 10 to 20 m based on the setup. Depending on whether a profile is currently measured or whether the sensors are sampling at a constant height, there is a temporal or vertical lag in the measurements of the two sensors that must be considered during calibration. We assume here that the mean profiles of the temperature and wind velocity do not change significantly over the short period of approximately 10 to 20 s needed for the lower sensor package to reach the former position of the upper sensor during ascent. A cross-correlation value is calculated for each profile to determine and remove the shift. With the data all at the same pressure level, a least-square fit can be obtained, and the calibration parameters can be determined. For constant-altitude measurements, we assume that the observations at the two heights of the probes are comparable in the calibration process, so we do not employ further corrections. The generally relatively stable conditions, especially during the polar night, support the validity of the stationarity assumption. However, to verify this in a qualitative manner, all calibrated profiles were manually compared to those measured by the tethersonde.

Finally, we provide two sets of calibrated velocity data: i) unfiltered wind velocities and ii) low-pass filtered data by applying a running median with a window size of 3 data points. The low-pass filter is used for two reasons: first, it minimizes the noise present in the data, which typically is observed at frequencies above approximately 20 Hz, and second, this type of filter very effectively eliminates individual spikes caused by droplet impact. The application of a complex filter algorithm to eliminate the influence of droplet impact^24^ can be omitted here due to the significantly low impact probability. Calibration of the thermocouple is easy relative to the hot-wire anemometer and is performed using a linear fit comparison to the temperature measurements of the tethersonde. The data are available with a temporal resolution of 10 Hz.

**Fig. 4:**
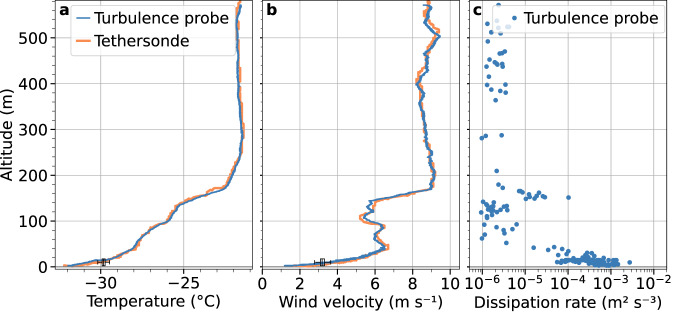
Profiles obtained by the turbulence probe on 25 January 2020. Panel (**a**) shows the air temperature, (**b**) the horizontal wind velocity, and (**c**) the energy dissipation rates. Also shown are the temperature (**a**) and wind velocity (**b**) profiles of the tethersonde used for calibration. For comparison, the Met Tower data measured at 10 m height of a 10 min time period (±5 min around the time when the balloon was at the lowermost level) is shown (box and whisker).

Figure 4 shows an example of the temperature and wind velocity profiles measured with the turbulence probe on 25 January 2020 (Panels a and b, respectively). Strong surface inversion is present with an increase in the wind velocity in the lowermost meters. The top of the inversion is located at approximately 170 m. In addition to the turbulence probe data, the temperature and wind velocity measured with the tethersonde are shown. The qualitatively favorable agreement between the two profiles provides confidence in the calibration procedure. The Met Tower data with a 10-minute time period around the time of balloon proximity to the surface are shown as a box-and-whisker plot. The temperature measurements of the turbulence probe are slightly below the range of the observed temperatures of the Met Tower at a 10 m height. The reason for this difference between the temperatures cannot be exactly identified. Both the turbulence probe and Met Tower wind velocity measurements agree well. Within this context, it is noted that the absolute accuracy of the wind determination imposes no primary influence on the relative resolution and, thus, on the turbulence measurements (refer to the *Technical Validation* section).

### Energy dissipation rate

**Fig. 5:**
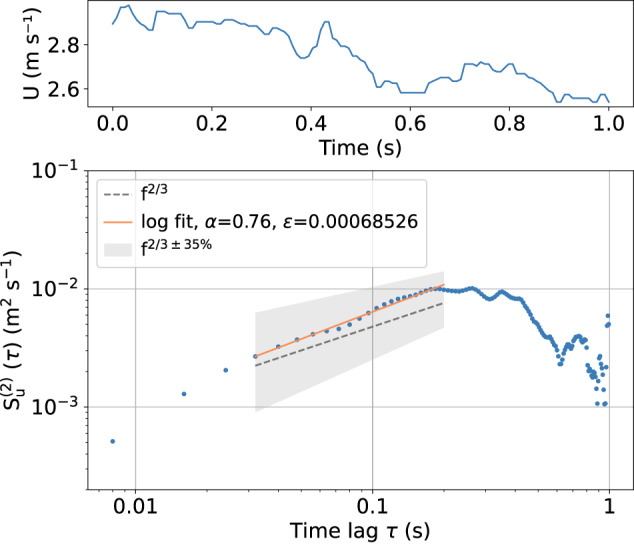
Horizontal wind velocity *U* of a time series of 1 s (upper panel) and the corresponding second-order structure function ${S}_{{\rm{u}}}^{(2)}$ (lower panel, blue) for a 1 s sub-record on 25 January 2020 during a descent. The inertial subrange scaling with *α* = 2/3 is indicated by the dashed line. The scaling of the sub-record (solid line, orange) is within the accepted range of *α* ±35% (shaded area) to derive the dissipation rate *ε*.

Local turbulence is often described with the (local) energy dissipation rate *ε*, i.e., is the rate at which turbulent kinetic energy is transferred from larger to smaller eddies in the inertial subrange until it is finally dissipated into heat^25,26^. A direct measure of *ε* requires velocity observations with a millimeter-scale resolution, which is challenging. Therefore, it is quite common to apply inertial subrange scaling. Here, we suggest the use of the scaling behavior of the second-order structure function to estimate *ε*, as this is a robust approach^27,28^:

3 $$\left\langle {\left(u(t+\tau )-u(t)\right)}^{2}\right\rangle =2{\left(\varepsilon \tau U\right)}^{\alpha }$$

with the scaling coefficient *α* = 2/3. The brackets < · > denote an average over a subrecord with a typical length on the order of one second, *τ* is the time lag, and *U* is the mean horizontal velocity of the subrecord, transforming time into length scales by applying Taylor’s frozen turbulence hypothesis. Figure 5 shows an example of the second-order structure function (lower panel) in a double logarithmic scale yielding a linear scaling with a slope of 0.76 close to the theoretical value of 2/3. The upper panel shows the wind velocity of the one-second subrecord.

This classical inertial subrange scaling approach is based on several assumptions, such as local isotropy and homogeneity, which may not necessarily be completely fulfilled in the atmosphere, especially under stable conditions. The more stable the stratification and the lower the turbulence, the more complicated the application of Kolmogorov’s similarity theory becomes^29^. The value of *α* will increasingly deviate from the theoretical value, and it becomes more difficult to observe an inertial subrange until the observations reach the noise level. Considering the abovementioned constraints, for data evaluation in this study, we consider a range for *α* of 2/3 ±35% as acceptable for *ε* estimates^14^. This threshold is somewhat arbitrary but should avoid misinterpretations in the calculation of *ε*. The estimated value of *α* is always stored in the datasets as a type of quality measure. Furthermore, the estimates of *ε* are based on the low-pass filtered data to minimize the influence of noise.

A second issue to be considered within this context is the width of the inertial subrange (Fig. 5, lower panel) used for estimating *ε*^30^. The outer length scale and, thus, the width of the inertial subrange depend on the stratification and the measurement height. Therefore, the range of the scales used for the linear fit to estimate *ε* is kept flexible and can be adapted to the conditions in each case. While the lower limit is set to 4/*f*_*s*_, which accounts for the low-pass filtering, the upper limits *τ* were empirically set to 0.8 s, 0.6 s, 0.4 s, and 0.2 s, thus excluding larger scale contributions. The algorithm can be used to calculate the four structure functions corresponding to the four inertial subranges and to perform linear fitting. The solution is employed to determine *ε* where the slope *α* is the closest to the theoretical value of 2/3. If *α* is not within the acceptable range for any of the time ranges, no *ε* value is calculated for the respective sample because turbulence cannot be resolved with the sensor. This flexible approach to define the inertial subrange and to derive *ε* allows for the estimation of *ε* over a wide range of conditions. The estimates of local energy dissipation rates are based on an averaging period of 1 s (125 sample points). This is a widely used averaging period in the literature for this type of calculation. The *ε* profile in Fig. 4c reveals that the estimation of local energy dissipation rates is possible even under very stable conditions with strong surface temperature inversion.

## Data Records

The data are stored as netCDF files separated into 47 sampling records measured on 34 days during the operation period of the turbulence probe. The postprocessed and calibrated data are published on PANGAEA^31^. Each file contains measured quantities as well as derived turbulence quantities, as listed in Table 1. Data variables with different sampling frequencies are merged along the time axis, and missing values are marked as “not a number”. The timestamp is given as YYYY-MM-DD HH:MM:SS.SSS. In the global metadata of each dataset, the tethersonde is specified, as three distinct tethersondes were used throughout the operation period. The metadata of each variable contain information on the units, instrument or method, platform, and long name. Furthermore, the percentage of derived energy dissipation rates during the sampling record can be found in the metadata. When no energy dissipation rate can be derived, the data are marked as “not a number”. Error-prone data, as occasionally observed in the temperature records, are cut off and replaced by “not a number”. The filename includes the date and time of the start of the measurements in the format YYYYMMDD_HHMM_UTC.nc.

**Table 1:** Data variables as stored in netCDF format. Long and short names are according to the meta data. Missing data due to different sampling frequencies *f*_*s*_ are filled with “not a number”.

Long name	short name	unit	*f*_*s*_
Time	time	UTC	125
Static pressure	p	hPa	1
Barometric height	zb	m	1
Temperature	T	°C	10
Horizontal wind speed	U	m s^−1^	125
Filtered horizontal wind speed	U_lp	m s^−1^	125
Local energy dissipation rate	epsilon	m^2^ s^−3^	1
Scaling coefficient	alpha	—	1
Inertial subrange upper limit	tau	s	1

## Technical Validation

### High-frequency wind velocity and energy dissipation rate

There are a few aspects to be considered by the user when interpreting wind velocity observations from a tethered balloon. One is related to balloon operation. Especially when starting and stopping the winch, forces are transferred to the balloon, which causes it to perform periodic movements. These movements influence the wind measurements but are easily detectable by eye. Another aspect concerns the balloon and its interaction with the (turbulent) wind field. Based on the experiences of Egerer *et al*.^14^, the balloon responds to the wind field with pendulum motions showing a typical frequency of approximately 0.5 Hz in the power spectral density (hereafter called the spectrum) of the measured wind velocity. However, such behavior occurs under convective conditions and is less pronounced under stable conditions. To verify this assumption, we analyze the spectra of the wind velocity as measured during a profile and a constant-altitude leg.

**Fig. 6:**
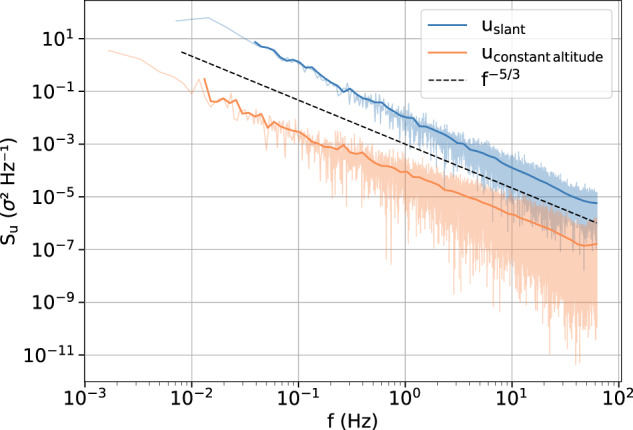
Power spectral density *S*_*u*_ of low-pass filtered wind measurements during a profile within the turbulent layer on 25 January 2020 between 12:36 and 12:39 UTC (blue). Additionally, the power spectral density of a time series with a constant height of 217 m, measured on 8 January 2020 between 07:49 and 07:59 UTC, is displayed (orange).

The spectrum measured on 25 January 2020 is shown in Fig. 6 (blue). This spectrum is based on a time series of approximately 140 seconds covering the lowermost 50 m of the descent of this flight, which occurs completely in the turbulent surface layer. Compared to Egerer *et al*.^14^ we did not observe any increased spectral densities due to pendulum motions of the balloon or the instrument. Therefore, the data were not filtered. Furthermore, a clear −5/3 slope was noted in the spectrum, indicating inertial subrange scaling. We could therefore conclude that the inertial subrange can be estimated with the given sensor and that local energy dissipation rates can be derived according to Eq. (3). The spectrum derived from the constant-altitude leg shows a gradual flattening at frequencies above 20 Hz, which indicates a spectral noise floor of *S*^(*n*)^≈2·10^−7^m^2^s^−1^. This value yields a minimum resolvable velocity difference^14^ of 3 mm s^−1^ and a minimum resolvable dissipation rate of ~10^−9^ m^2^ s^−3^ as a lower limit (Fig. 4c). This estimate depends on the mean wind velocity (5 m s^−1^ here) due to the application of Taylor’s hypothesis. In addition, the slope criterion ensures that these values meet the quality requirements.

When a turbulent parameter such as the local energy dissipation rate is determined based on an averaging process, there is always the fundamental conflict between averaging over a short time to resolve small-scale structures and averaging over a long enough time to achieve statistical significance^32^. The error of a single estimate of *ε* depends on the number of spectral points *N* above the noise level within the averaging window and can be given as $1/\sqrt{N}$^18^. For example, the number of points in the shaded area in Fig. 5 can be used to estimate the slope of the structure function, and thus *ε* is *N* = 22, yielding an error of 20%.

A quantitative estimate of how many *ε*-profile measurement realizations are needed to obtain a robust mean profile goes beyond this discussion and can be found in the literature^33^. However, to assess how representative the individual vertical profiles are in general, it is advisable to consider the Met Tower data at 10 m in parallel over the period of the balloon measurements. During the descent shown in Fig. 4, for example, the horizontal velocity decreased almost linearly from 4 to 3 m s^−1^, but after subtracting the trend, the fluctuations were uniformly distributed at *σ*_*u*_ = 0.1 m s^−1^. This is not a quantitative measure of the representativeness of a single profile, but it can indicate the extent to which the turbulence measured at the balloon is only a short snapshot or can be considered valid for a certain period of time.

Finally, isolated cloud droplets (or ice particles) impacting the hot wire cause large spikes in the dataset due to rapid evaporation. We determined energy dissipation rates from both unfiltered velocity data and data subjected to a median filter. On the one hand, the filter reduced the magnitude of the energy dissipation rates by up to 30% for individual data points. On the other hand, more data points now passed the quality control test, and dissipation rates could be determined in less turbulent regions. The background for this approach is that before filtering, the slope of the linear fit in the inertial region was slightly reduced under the influence of the noisy, high-frequency part in the spectrum (or in the structure function). This led to overestimation of *ε* and partly to the exclusion of values since the slope was no longer acceptable. A reduction in the values by 30% is remarkable at first sight, but these values already partly comprise results with different methods for determining *ε*^33^. Furthermore, one must consider that this parameter is lognormally distributed in space and time. Notably, the structure of a given *ε* profile does not significantly change with filter application, but we can safely conclude that low-pass filtering leads to more robust and reliable dissipation data.

### High-frequency temperature

**Fig. 7:**
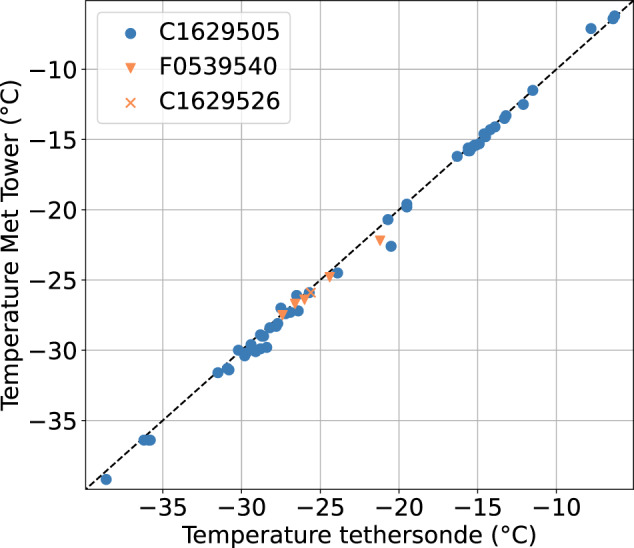
Comparison of temperature measurements of the tethersonde C1629505 (blue) at 10 m with 10 m temperature measurements of the Met Tower. Further, the 10 m temperature is shown for two other tethersondes, F0539540 and C1629526, which were used during the operational period.

During legs 1, 2, and 3, three distinct tethersondes were in use. The majority of the flights were performed with the tethersonde C1629505, five flights were conducted with the tethersonde F0539540, and one flight was conducted with the tethersonde C1629526. To better assess the quality of the tethersonde temperature measurements, the measurements at a height of 10 m on the balloon were compared to the Met Tower measurements and cross plotted (Fig. 7). The Met Tower observations are considered representative because the sea ice cover remained mostly homogeneous in winter and only minor ice dynamics occurred^12^.

Over the entire measurement period, there is a high range of measured temperatures at 10 m, so we can obtain robust estimates of the offset and slope using linear regression. Since the deviation in the slope from one is negligible, we can further assume an offset value of 0.2 K as the absolute accuracy of the C1629505 tethersonde temperature measurements. Due to the limited number of flights involving tethersondes F0539540 and C1629526, individual comparison of these tethersondes was impossible over a sufficient range of temperature values. Therefore, the calibration of C1629505 was applied, and the datasets contain the number of the respective tethersonde in the metadata.

In 27 out of 98 profiles, we observed an unusual temperature behavior shortly after the launch, which could probably be explained by the large differences between the temperatures in the tent (probe preparation) and the (low) outside temperatures. Although the short response time of the thermocouple is sufficient to measure this temperature drop, this misbehavior is reproducible and only observed during the first ascent of a given flight. The reason is most likely that the internal temperature sensor, which is used to measure the temperature at the thermocouple compensation point, is too slow to adequately measure this temperature jump. We removed these data from the dataset after manual review and marked them as “not a number”.

## Data Availability

The algorithm for the calculation of energy dissipation rates was written in Python. It was developed for the described turbulence records and is freely available on Zenodo^34^.
